# A Flexible Bayesian Model for Studying Gene–Environment Interaction

**DOI:** 10.1371/journal.pgen.1002482

**Published:** 2012-01-26

**Authors:** Kai Yu, Sholom Wacholder, William Wheeler, Zhaoming Wang, Neil Caporaso, Maria Teresa Landi, Faming Liang

**Affiliations:** 1 Division of Cancer Epidemiology and Genetics, National Cancer Institute, Rockville, Maryland, United States of America; 2 Information Management Services, Rockville, Maryland, United States of America; 3 Core Genotyping Facility, SAIC Frederick, National Cancer Institute–Frederick, Frederick, Maryland, United States of America; 4 Department of Statistics, Texas A&M University, College Station, Texas, United States of America; University of California San Diego and The Scripps Research Institute, United States of America

## Abstract

An important follow-up step after genetic markers are found to be associated with a disease outcome is a more detailed analysis investigating how the implicated gene or chromosomal region and an established environment risk factor interact to influence the disease risk. The standard approach to this study of gene–environment interaction considers one genetic marker at a time and therefore could misrepresent and underestimate the genetic contribution to the joint effect when one or more functional loci, some of which might not be genotyped, exist in the region and interact with the environment risk factor in a complex way. We develop a more global approach based on a Bayesian model that uses a latent genetic profile variable to capture all of the genetic variation in the entire targeted region and allows the environment effect to vary across different genetic profile categories. We also propose a resampling-based test derived from the developed Bayesian model for the detection of gene–environment interaction. Using data collected in the Environment and Genetics in Lung Cancer Etiology (EAGLE) study, we apply the Bayesian model to evaluate the joint effect of smoking intensity and genetic variants in the 15q25.1 region, which contains a cluster of nicotinic acetylcholine receptor genes and has been shown to be associated with both lung cancer and smoking behavior. We find evidence for gene–environment interaction (P-value = 0.016), with the smoking effect appearing to be stronger in subjects with a genetic profile associated with a higher lung cancer risk; the conventional test of gene–environment interaction based on the single-marker approach is far from significant.

## Author Summary

Many common diseases result from a complex interplay of genetic and environmental risk factors. It is important to study the potential genetic and environmental risk factors jointly in order to achieve a better understanding of the mechanisms underlying disease development. The standard single-marker approach that studies the environmental risk factor and one genetic marker at a time could misrepresent the gene–environment interaction, as the single genetic marker might not be an appropriate surrogate for the underlying genetic functioning polymorphisms. We propose a method to look at gene–environment interaction at the gene/region level by integrating information observed on multiple genetic markers within the selected gene/region with measures of environmental exposure. Using data collected in the Environment and Genetics in Lung Cancer Etiology (EAGLE) study, we apply the proposed model to evaluate the joint effect of smoking intensity and genetic variants in the 15q25.1 region and find evidence for gene–environment interaction (P-value = 0.016), with the smoking effect varying according to a subject's genetic profile.

## Introduction

Genome-wide association studies that focus on detecting the main effect from individual single nucleotide polymorphisms (SNPs) have successfully identified more than 4,000 SNPs associated with different diseases [1]. To achieve a better understanding of the mechanisms underlying disease development, it is of great interest to follow up those genetic findings with more detailed analyses investigating how the gene and environment interact in their influence on disease risk. One popular approach aims at detecting SNP-environment interaction between individual SNPs and established environmental risk factors [2], [3], [4]. One of the few successes for this approach is the interaction detected between cigarette smoking and two genetic variants, a NAT2 tagging SNP and a GSTM1 deletion, in a multi-stage genome-wide association study (GWAS) of bladder cancer [3].

The standard approach to the study of gene–environment joint effect inspects one marker at a time, assuming that a single marker is the functional unit in the gene and environment interplay. This single-marker approach could misrepresent and underestimate the genetic contribution to the joint effect when one or more functional loci, some of which might not be genotyped, exist in the region, and interact with the environment risk factor in a complex way. A more global approach that simultaneously considers all genetic markers might capture more of the genetic variation within the entire targeted region, and provides a better opportunity to reveal complicated gene–environment interactions [5]. The global approach would be more informative if it has the capability showing how an environmental effect varies according to a subject's genetic profile.

We provide a flexible Bayesian modeling framework for the study of gene–environment joint effects. We consider a case-control study with genotypes **G** at a set of SNPs within a given region and a measurement for the environment exposure *E* available for each subject. We seek to identify a latent genetic profile variable *L* that classifies the multilocus genotype **G** into different categories (clusters) such that subjects with their genotype assigned to the same genetic profile category share the same disease risk model, which is a standard logistic regression model with its own intercept term and slope. The intercept term represents the baseline log odds, common for subjects sharing the same genetic profile. The slope represents the effect (i.e., log odds ratio) of the environment risk factor for subjects with the given genetic profile. The model that we try to build and make inferences from is essentially the logistic regression model consisting of *L* and *E* as main effects and their product as an interaction term; the unusual aspect is that the definition of the latent genetic profile *L* is a priori unknown. To account for the uncertainty in the cluster assignment underlying the definition of *L*, we adopt an idea from the hidden Markov model originally developed for modeling the spatial heterogeneity of the disease event rate, observed on a predefined set of areas [6]. In this Bayesian model approach, Green and Richardson tried to allocate areas into a number of clusters and assumed a common disease rate for areas assigned to the same cluster. The mechanism for the area allocation was modeled through the Potts model [7], which favors probabilistically those allocation patterns where “neighboring” areas are assigned to the same cluster. Note that the spatial dependence assumption is generally appropriate in situations where the event rate is expected to take on similar values in neighboring areas. To draw the connection, we can think of each type of observed multi-locus genotype **G** as an “area”. We would like to use the Potts model to guide the cluster assignment through a certain level of “spatial” dependence, i.e., similar genotypes (nearby areas) tend to be assigned to the same cluster, as in other applications in genetics studies, including the study of haplotype association [8], [9].

We use the Markov chain Monte Carlo (MCMC) sampling method (e.g., [10], [11]) to fit the proposed model, incorporating several recent advances in the MCMC methodology. We adopt a recently developed algorithm [12] to update the regulating parameter in the Potts model, which has an intractable normalizing constant, and cannot be handled by the standard Metropolis Hastings algorithm. This algorithm allows us to consider the parameter of interest on its original continuous scale and obviates the need for a finite number of selected grids with their normalizing constants pre-calculated, a strategy taken by Green and Richardson [6]. To identify the optimal genetic profile assignment, we use an ensemble averaging method that aggregates different cluster assignments generated by the MCMC samplers into a consensual one. We find that this cluster algorithm works quite well in simulation studies. A similar idea has been used by Liang [13] and Molitor *et al* [14] in different contexts. We also propose a resampling-based test based on the fitted Bayesian model that can be used to formally test for the existence of gene–environment interaction.

We apply the proposed method to study the joint effect of cigarette smoking intensity and genetic variants in chromosome region 15q25.1 using data from EAGLE, a population-based case-control study conducted in Italy [15]. Cigarette smoking is an established major risk factor for lung cancer. Besides environmental exposures, recent GWAS identified a few chromosome regions (e.g., chromosomes 15q25.1, 5p15, and 6p21) harboring genetic variants underlying a susceptibility for lung cancer [15], [16], [17]. In particular, the chromosome 15q25.1 region, which includes the *CHRNA5-CHRNA3-CHRNB4* cluster of cholinergic nicotinic receptor subunit genes, has been shown to be associated with both lung cancer and smoking behaviors, such as cigarette smoking intensity [18], [19], [20], [21], [22]. Although there is no evidence suggesting the existence of multiple loci in this region independently contributing to lung cancer susceptibility in populations of European ancestry [16], it does appear that there are multiple independent loci within 15q25.1 affecting smoking intensity [19]. The main goal of our analysis is to evaluate whether the effect of smoking intensity varies with genetic variants in 15q25.1. Our analysis finds evidence for gene–environment interaction, with the relative risk for smoking appearing to be stronger in subjects with a genetic profile associated with a higher lung cancer risk. The proposed resampling-based test derived from the fitted Bayesian model also detects significant gene–environment interaction (P-value = 0.016). On the other hand, the standard single-marker approach that aims at detecting the interaction between a SNP and smoking intensity fails to reveal any evidence of interaction, with the smallest observed nominal P-value being 0.021 among the 32 testing SNPs, and the adjusted P-value based on the permutation test being 0.29.

## Materials and Methods

We first introduce the Bayesian model and describe the MCMC algorithm for fitting this model. Next we provide procedures for posterior inference using samples generated by the MCMC sampler, including a method for deciding the number of clusters and a method for identifying the optimal cluster assignment once the number of clusters is determined. We validate the proposed method using simulated data. We then apply the method to study the gene–environment joint effect using data generated from the EAGLE lung cancer case-control study.

### The Bayesian Model Setup

Assume we have data collected from a case-control study, with 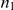 cases, 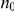 controls. Let 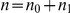 be the total number of subjects in the study. For the *i*th subject, we denote its observation by 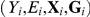, where 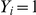 for a case, 0 for a control; 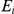 is the observed exposure for the environment risk factor of interest; 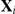 represents measures on a set of covariates; and 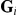 represents multilocus genotypes observed on a set of SNPs in a pre-specified region. In the following discussion, we use the term genotype to refer to the multilocus genotype observed on all considered SNPs within the targeted region. We intend to develop a model for the **G**-*E* joint effect that permits **G**-*E* interaction. More specifically, we assume the true underlying risk model has the following form:
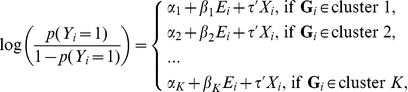
(1)where clusters 1 to *K* represent a partition of the genotype space; 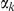 is the intercept term representing the common baseline log odds for subjects with their genotypes in cluster *k*; 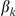 is the effect of *E* (in term of log odds ratio) in the disease model for cluster *k*, 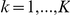; and 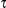 is the vector of coefficients for the set of covariates *X* and is constant regardless of a subject's genotype. Notice that if the partition of the joint genotype space is known a priori, we can derive the corresponding *K*-category genetic profile variable *L* based on the cluster assignment. The above model (1) is then essentially the standard logistic regression model consisting of *L* and *E* as main effects and their product as the interaction term, with adjustment for *X*, and has the following form:
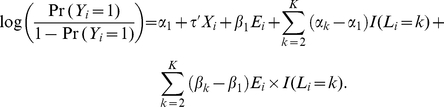
Thus it is clear that there is no **G**-*E* interaction if 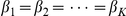, and the interaction exists if otherwise.

In real applications, we do not know a priori the partition of the genotype space. If **G** consists of just one SNP, the goal can be achieved easily by using a saturated logistic regression including both *E* and **G** (as a three-level categorical variable) as the main effects and their product as the interaction term. For situations where **G** consists of multiple SNPs (e.g., more than 10), as in the case of the EAGLE lung cancer study, we propose the following Bayesian model that simultaneously searches for the optimal partition of the genotype space and estimates the unknown parameters in the corresponding risk model (1).

The Bayesian model is built up in a hierarchic framework. We first describe our model by assuming *K*, the total number of clusters, is known. We will describe how to choose *K* later. Suppose there are *H* types of genotype configurations observed in the sample, labeled as genotype 1, 2, …, *H*. We define the latent genotype allocation vector 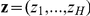, with 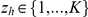, being the cluster assignment for genotype *h*, 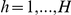. For subject *i*, we denote its genotype id by 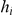. Given the allocation vector 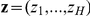 and the set of coefficients 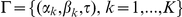 for the disease model (1), the probability of subject *i* having the disease outcome is
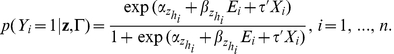
(2)


In the above model specification, we use the prospective likelihood function (2) for observed case-control data, which were collected under a retrospective sampling scheme given the disease outcome. The use of the prospective likelihood function can be partially justified by the general results from Staicu [23] and Seaman and Richardson [24]. They showed the equivalence of prospective and retrospective analysis in the Bayesian framework in the sense that both approaches could yield the identical marginal posterior distribution of the log odds ratio under analyses with properly specified priors. In model (2), the effect of *E* varies with **G**. Thus we call it the Bayesian risk model allowing for **G**-*E* interaction. As a comparison in the analysis, we also consider a model assuming a homogeneous effect from *E*, which is defined as
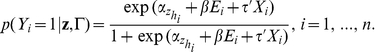
(3)We call this model the Bayesian risk model without **G**-*E* interaction. In what follows, we will describe methods for fitting model (2), the one allowing for **G**-*E* interaction. Similar procedures can be applied to model (3).

To model the distribution of the allocation vector **z**, we first choose a similarity metric to define the spatial contiguity between any two genotypes. Let *J* denote the total number of considered SNPs within the region, with the genotype at a given SNP being coded as 0, 1, or 2 according to the number of copies of the minor allele. Let genotypes *h* and 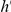 have the configurations 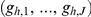 and 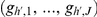, where 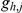 is the genotype at the *j*th SNP for the multilocus genotype *h*. We first define the distance between them as
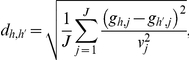
where 
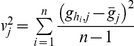
 is the variance for the genotype at SNP *j* observed in the sample, with 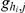 being the genotype at SNP *j* for subject *i*, and 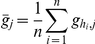. Then we define 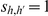 if 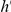 is among the 4 (distinctive) genotypes closest to genotype *h*, and 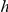 is among the 4 genotypes closest to genotype 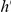; 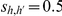 if 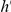 (or *h*) is among the 4 genotypes closest to genotype *h* (or 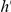) but this is not true in both cases; and 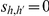 for all other cases.

We model **z** with the Potts model, which has a regulating parameter 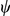 governing the level of spatial dependency in the cluster assignment. The Potts model has the following form:
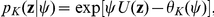
where 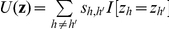, with 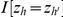 being the indictor function, i.e., 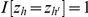 if 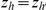 and 0 otherwise, and where
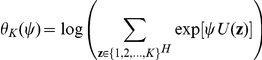
is the log normalizing constant. Under the Potts model with 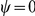, the cluster assignments are allocated independently for different genotypes. When 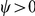, the cluster assignments for two neighboring genotypes *h* and 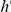 (i.e., two genotypes with 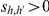) are correlated. The level of correlation (spatial dependence) increases with 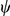. For example, under the genotype configuration observed in the EAGLE study and 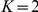, the average probability that any two neighboring genotypes are allocated to the same cluster is 0.5 when 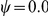. It increases to 0.83, and 0.97 for 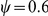 and 1.2, respectively. More discussions of the Potts model can be found in [6].

We need to specify our prior models for 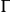 and 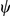. In this paper, we consider the normal distribution with a mean of 0 and a variance of 4 or the uniform distribution on the interval of 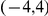 as the prior for each parameter in 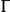. We describe the appropriateness of those priors for the prospective likelihood model in the Discussion Section. Both priors are very uninformative and generate similar conclusions on the EAGLE study and simulated datasets. Therefore we present only results based on the normal prior in the following discussions. Following Green and Richardson [6], the prior distribution 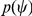 for 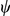 is set to be a uniform distribution on the interval 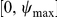, which covers an appropriate region of 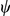 such that the resulting class of Potts models are flexible enough to capture a wide range of spatial dependence. We note that 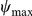 cannot be too large. If 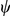 is over a critical point, the corresponding Potts model would essentially force almost all elements into the same cluster, a well known phenomenon for the Potts model called phase transition property [25], and in this situation, the MCMC simulation tends to get stuck. We did some experiments to explore the setting of 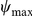 for the Potts model based on the neighborhood configuration observed in the EAGLE study. We found the value 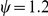 induces a high level of spatial dependence, with the average probability that any two neighboring genotypes are allocated to the same cluster being 0.97 at 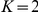; and when 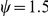, the average probability goes to 0.99, which indicates an extremely high level of spatial dependence for the Potts model. Based on these observations, we decided to set 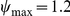 in our EAGLE study application, as well as in simulation studies that assume the same neighborhood structure as the EAGLE study. We consider only a uniform prior for 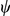 since in practice we usually do not know which level of spatial dependence is more likely than the others. But the algorithm described below can certainly be used with other prior functions if necessary.

Putting all the foregoing models together, we can express the joint distribution of all variables as
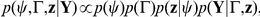
where 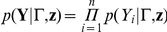. The inference (for a fixed total number of clusters *K*) on 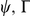, and **z** can be based on the following MCMC algorithm.

### The MCMC Algorithm

#### Updating coefficients 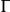


The full conditional function for coefficients 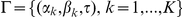 in the risk model can be written as
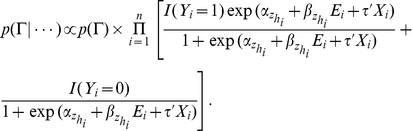
(4)We can use the standard Metropolis-Hastings (MH) steps to update 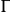, conditioned on the current values of other parameters. The detailed algorithm is given in Text S1.

#### Updating the allocation vector 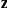


Following Green and Richardson [6], we can update the allocations **z** using a Gibbs kernel; that is, for the genotype *h*, its cluster assignment is updated by drawing from the following full conditional distribution,
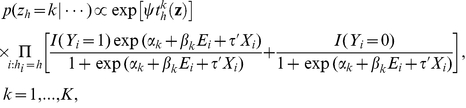
(5)where 
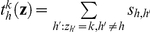
 is the sum of similarity scores between the genotype *h* and other genotypes currently assigned to cluster *k*.

Since the sampling space for **z** is discrete, the standard Gibbs sampler can be improved by the Metropolized Gibbs sampler [26]. Thus we choose this sampler for updating the allocation vector. A summary of the algorithm is given in Text S1.

#### Updating the regulating parameter 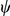


The regulating parameter 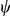 has the following full conditional distribution:
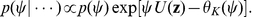
(6)If the standard MH algorithm is used, updating 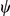 would involve the evaluation of the normalizing constant 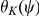 for the Potts model, which is prohibitive when the dimension of **z** is large. Green and Richardson [6] chose to restrict 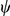 to a pre-specified finite set of values; they used the thermodynamic integration approach [27] to estimate 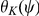 for a given value of *K*. Those estimates were then used in the MCMC sampler. The estimate of 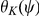 at pre-specified grid points might lead to biased Monte Carlo estimates of 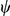 and other parameters.

Here we propose to use the recently developed Monte Carlo Metropolis-Hastings algorithm (MCMH) [12] to sample 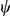 from 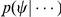. This new algorithm replaces the ratio of normalizing constants at any two values of 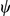 by a Monte Carlo estimate, which is obtained through a set of *m* auxiliary samples, in the MCMC iterations, thus allowing us to consider 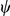 on its original continuous scale instead of on a finite number of pre-specified points. As shown in [12], this algorithm ensures that the Monte Carlo estimate of the parameter will converge to its posterior mean. In our numeric experiments, we find it is appropriate to choose the number of auxiliary samples *m* to be between 50 and 100. A summary of the algorithm for updating 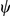 is given in Text S1.

### Posterior Inference

In our simulation studies and the real data application, we find the MCMC algorithm generally converges after 100.000 iterations. Below we describe a procedure for determining the number of clusters, and an ensemble averaging method for the identification of the cluster assignment based on the MCMC samples.

#### Determining the number of clusters

We choose to use the deviance information criterion (DIC) proposed by Spiegelhalter *et al* [28] for determining the number of clusters. For a given number of clusters *K*, define the deviance 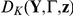 as
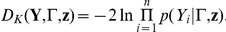
We can calculate the posterior expected deviance 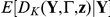 by averaging the deviance calculated at samples of 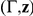 generated by MCMC output. We calculate the deviance 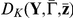 at the posterior mean of the parameters as
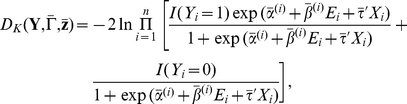
where 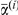 and 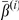 are the posterior means of the coefficients assigned to subject *i*; 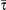 is the posterior mean for 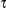. The 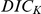 for the model with *K* clusters is then calculated as
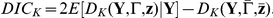



To determine the number of clusters, we run the algorithm with different values of *K* (e.g., 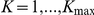, with 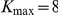) and compute their DIC values. The DIC criterion favors models with small DIC values. To take the Monte Carlo variation into the consideration, instead of choosing the *K* with the smallest DIC, we adopt the +1 standard error (SE) rule originally proposed for the tree model selection [29]. To use this rule, we run the MCMC algorithm 20 times, with different random seeds for each considered value of *K*, and then pick the optimal number of clusters 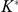 as the smallest one such that
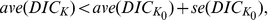
(7)where 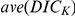 is the average of the values of DIC measured at *K* over 20 runs, 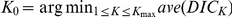, and 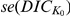 is the Monte Carlo standard error estimated for 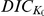 based on 20 runs.

Based on our numerical experiments, we found that the Monte Carlo standard error usually is less than 1 if the MCMC chain converges. So, if there is only one run for each *K*, we recommend picking 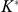 as the smallest one such that
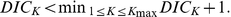
(8)We use this rule, hereafter called the +1 rule, to select the optimal number of clusters in simulation studies.

#### Identifying the cluster assignment

After the determination of the number of clusters 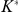, it is usually helpful to identify the consensual cluster assignment rule that assigns each genotype to one of the 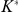 clusters. We can also use this partition to assign each subject to one of the clusters based on his or her genotype's assignment. Here we adopt the ideas from Liang [13] and Molitor *et al* [14] to find such a partition. Based on the samples generated from MCMC runs under the 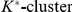 model, we let 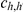 be the proportion of times that the genotypes *h* and 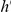 are assigned to the same cluster. We then use 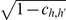 as the dissimilarity metric and apply the PAM (partitioning around medoids) method [30] to partition genotypes into 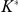 clusters. Simulation studies presented later show this clustering algorithm works quite well in identifying the appropriate clusters.

### A Resampling-Based Test for Gene–Environment Interaction

It is usually desirable to have a formal statistical test or decision rule for inference regarding the presence of an interaction. Here we propose a resampling-based test for this purpose. First we fit model (2), the Bayesian risk model allowing for **G**-*E* interaction, under various numbers of clusters. Then we use the +1 rule to identify 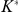, the optimal number of clusters that is not less than 2, and the corresponding consensual cluster assignment *L*. We require 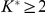 for this interaction test because the interaction test is not defined for 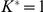. We use the maximum likelihood estimate (MLE) to establish the following logistic regression model,
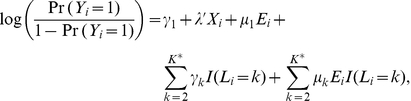
(9)where 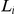 is the cluster assignment for subject 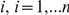, given by the consensual cluster assignment *L*. This model includes the main effects of *L* and *E*, as well as their interactions. We can conduct a likelihood ratio test comparing model (9) with the similar model without the interaction terms and obtain the corresponding “P-value”, denoted by 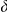, based on the Chi-squared distribution with 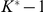 degrees of freedom (df). Clearly, this “P-value” 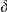 tends to overestimate the significance level of the interaction, as the variable 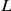 is data-driven, but a small value for 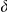 provides evidence against the null. We can use 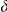 as the test statistic and apply the following resampling-based procedure to evaluate its significance level.

1. Apply the MCMC procedure to fit model (3), the Bayesian risk model without **G**-*E* interaction, on the observed data and identify 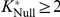, the optimal number of clusters, using the +1 rule, as well as the corresponding consensual cluster assignment.
2. Use MLE to fit the following logistic regression model based on the observed data,
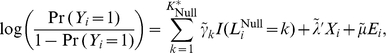
(10)where 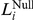 is the cluster assignment for subject 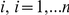, given by the consensual cluster assignment identified in Step 1, and 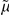, 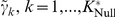, and 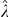 are the estimated coefficients.
3. Use the model given by (10) to generate *B* sets of bootstrap null datasets. Each null dataset is a copy of the observed dataset, except the outcome for every subject is regenerated according to the probability model given by (10).
4. For the *b*th null dataset, 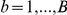, obtain the test statistic 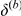 using the same procedure used above for obtaining 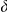.
5. The estimated P-value for 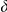 is given by 
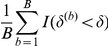

In Steps 1 and 2 we establish the Bayesian risk model under the null hypothesis that there is no **G-** *E* interaction and the corresponding logistic regression model. We use the fitted logistic regression model (10) to generate multiple null datasets in Step 3 based on the parametric bootstrap procedure [31]. In Step 4, for the *b*th generated null dataset, we first apply the MCMC procedure to establish the Bayesian model given by (2) and next identify the optimal number of clusters with the +1 rule, as well as the corresponding consensual cluster assignment. Then we fit the corresponding logistic regression model with **G-** *E* interaction and obtain the test statistic 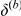 from the likelihood ratio test.

## Results

### The EAGLE Study

We used data generated by the lung cancer GWAS in the EAGLE study [15] with 1920 lung cancer cases and 1979 population controls as the basis for our simulation studies and real data applications. We focused on the chromosome region 15q25.1 between 76.5 Mb and 76.72 Mb, with the boundary defined by loci where the recombination rate is relatively high. This region covers all replicated loci relating to smoking behavior or lung cancer risk. We have genotypes on 32 SNPs in the region that have a minor allele frequency (MAF) larger than 4% (estimated in 1979 EAGLE control samples). We removed 17 redundant SNPs, leaving a minimal set of 15 SNPs where the pairwise 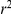 was always less than 0.8. We used genotypes on these 15 tagging SNPs to represent each subject's genetic variation pattern in the region. The reason for removing redundant SNPs is to ensure that the similarity measure between any two types of multilocus genotypes is not dominated by a set of SNPs in high linkage disequilibrium. The summary of the 15 chosen tagging SNPs is given in Table 1.

**Table 1:** Summary of 15 tagging SNPs chosen for the EAGLE study.

SNP id	Position	MAF^a^	Odds ratio^b^	P-value^b^	PC1^c^	PC2^c^
rs1394371	76511524	0.32	1.110685	7.34E-02	0.238102	***0.289431***
rs12903150	76511700	0.22	0.916667	1.89E-01	−0.22088	0.066403
rs12899131	76513940	0.38	0.973222	6.27E-01	−0.36255	0.013854
rs2656069	76532762	0.23	0.760253	7.67E-05	0.110054	***−0.40066***
rs13180	76576543	0.39	0.873685	1.75E-02	−0.08845	***−0.374***
rs3743079	76578116	0.17	1.075484	3.13E-01	−0.2273	−0.05414
rs3885951	76612972	0.12	1.279568	2.30E-03	0.158959	***0.186958***
rs2036534	76614003	0.24	0.698668	1.23E-07	0.120671	***−0.42301***
rs2292117	76621744	0.36	0.908155	8.78E-02	−0.39076	0.034655
rs680244	76658343	0.37	0.911216	1.03E-01	−0.39566	0.037326
rs578776	76675455	0.29	0.738028	1.45E-06	0.085806	***−0.39304***
rs12914385	76685778	0.41	1.416705	3.68E-10	0.258519	***0.31863***
rs1948	76704454	0.3	0.878962	3.10E-02	−0.36327	0.013799
rs11636753	76716001	0.35	0.89959	6.74E-02	−0.35628	0.025404
rs12441998	76716427	0.24	0.725298	2.24E-06	0.096535	***−0.36738***

a The minor allele frequency was estimated based on the control samples in the EAGLE study.

b The per-allele OR and the one degree of freedom Wald test for the association between lung cancer and the SNP based on the logistic regression model adjusted for smoking intensity, age, and gender. The SNP genotype was coded as the copy number of the minor alleles.

c Loadings of individual SNPs on the first and second principal components based on the principal component analysis conducted on the selected subjects from the EAGLE study used in the real-data application. The highlighted values in the PC2 column are the ones that dominate the definition of the 2^nd^ principal component.

### Simulation Studies: Performance of the Bayesian Model

**Figure 1:**
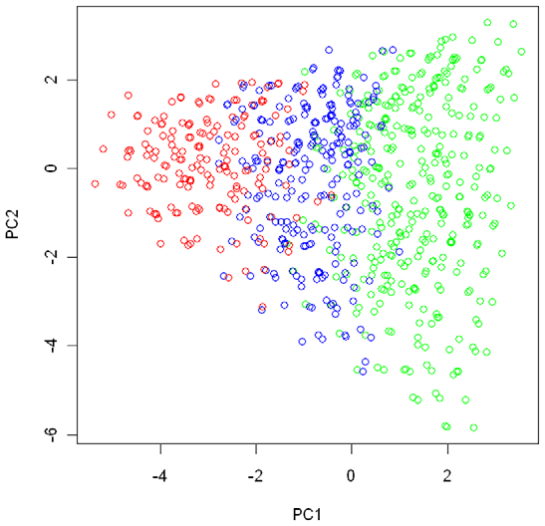
The partition of the genotype space in the simulation study. We conducted a principal component analysis on all subjects from the EAGLE study with genotypes at the 15 chosen tagging SNPs as coordinates. We plot subjects by their first and second principal components. Subjects with the same multilocus genotype were represented by a single point in the plot. The points in green, blue, and red colors are those subjects (genotypes) belonging to region I (consisting of genotypes having no more than 1 risk allele among the three considered functional SNPs), region II (consisting of genotypes having 2 risk alleles), and region III (consisting of genotypes have more than 2 risk alleles).

We conducted simulation studies to evaluate the performance of the proposed method for fitting the Bayesian model allowing for **G**-*E* interaction. In the simulation study we were interested in studying the interaction between a binary environment risk factor (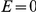 or 1) and genetic variants (**G**) within a candidate region. We used genotypes at 15 tagging SNPs (Table 1) in 15q25.1 observed in the EAGLE study to represent the joint genotype distribution for the simulated population, which consisted of 766 distinct multilocus genotypes. We chose the 2^nd^, 6^th^, and 10^th^ SNPs listed in the Table 1 as the functional SNPs, and divided the genotype space into the following three regions according to the total number of risk alleles (assuming the minor allele to be the high-risk allele) among the 3 functional SNPs: region I, consisting of genotypes with 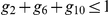; regions II, consisting of genotypes with 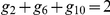; and region III, including genotypes with 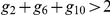. We conducted a principal component (PC) analysis on subjects from the EAGLE study with genotypes at the 15 SNPs as their coordinates. Figure 1 shows how genotypes (subjects) in each of the three regions were distributed in the first 2-PC space, with regions I, II, and III in green, blue, and red, respectively.

The disease risk models we considered had the form given by (1). Their definitions are given in Table 2. Under Model 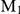 there was no genetic effect and no interaction between **G** and *E*, and thus there was no risk heterogeneity in the genotype space. Under 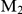 and 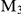, coefficients 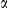 and 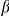 had the same clustering pattern. Under models 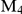, the risk heterogeneity patterns for 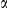 and 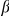 were not matched, unlike those under model 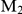 and 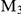. In model 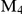, the two clusters defined by 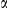 were region I, and regions II and III combined, while the two clusters defined by the effect of 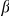 were regions I and II combined, and region III.

**Table 2:** List of disease risk models considered in the simulation study for evaluating the Bayesian model.

Model id	Coefficients^a^	Cluster 1^b^	Cluster 2^b^	Cluster 3^b^
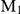	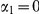 , 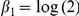	No restriction	NA	NA
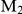	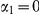 , 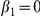 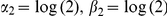	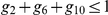	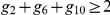	NA
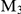	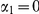 , 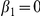 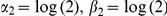 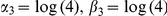	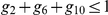	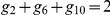	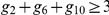
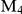	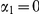 , 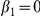 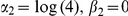 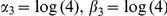	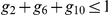	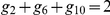	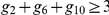

a The coefficients are defined for models with the form given by (1) in the main text.

b The cluster is defined according to the total number of risk alleles at the three chosen SNPs (the 2^nd^, 6^th^, and 10^th^ SNPs listed in Table 1).

We assumed that the environmental exposure status *E* (0 or 1) and **G** were correlated in the general population. The distribution of *E* depended on **G** in the following way: for a subject with genotype in region I, the probabilities of being unexposed (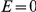) or exposed (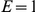) were 0.7 and 0.3; for a subject with genotype in one of the other two regions, those probabilities were 0.4 and 0.6 for 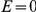 and 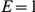. Thus the distribution of *E* was quite different for subjects with different genotypes.

Under each model, we simulated 50 datasets representing a case-control study with 1500 cases and 1500 controls. We ran the MCMC algorithm with 2,000,000 iterations with the first 1,000,000 iterations being discarded. We used an algorithm similar to that described in [32] to simulate the case-control study. Note that under the case-control sampling scheme, we do not need to specify a value for 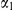. Instead, we just need to know the values of 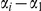, 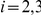, in order to simulate datasets from a case-control study.

For each simulated dataset, we applied our method with 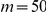 auxiliary samples, with the number of clusters *K* ranging from 1 to 8. We used the +1 rule defined by (8) to identify 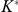, the optimal number of clusters. Table 3 provides a summary of the number of clusters identified over 50 simulated datasets under each risk model. We can see from the table that the +1 rule performs quite well in identifying the right number of clusters, even in situations where there is no clustering structure (i.e., the true number of clusters, 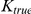, is 1).

**Table 3:** Performance of the +1 rule for identifying the number of clusters in the simulation study.

	Total number of clusters identified ( 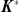 )				
Model id	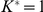	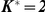	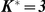	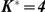	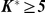
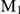	***48***			1	1
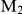		***46***	3		1
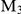			***45***	5	
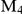			***42***	7	1

There are 50 simulated datasets under each model. The counts are the frequencies for the number of clusters identified by the +1 rule defined in the main text. The highlighted counts are the number of times the algorithm identified the correct number of clusters.

We evaluated the performance of the algorithm for cluster assignment by comparing the cluster assignment estimated at 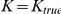 with the true underlying cluster assignment chosen by the simulation design. For model 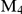, the clustering patterns for 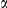 and 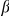 were not matched. In this case we treated the finer partitioning (given by Figure 1) that accommodates the clustering patterns of both 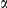 and 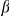 as the true one. The accuracy of the estimated cluster assignment was measured as the proportion of subjects being assigned to the same cluster by both assignments (the estimated one and the true one). The accuracy summary over 50 replications under various considered models (except 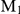, the model with no clustering structure) is given in Table 4. It indicates that the cluster assignment algorithm appears to be able to partition the subjects (and genotypes) into the proper subgroups, provided that the correct number of clusters can be identified.

**Table 4:** Performance of the algorithm for the cluster assignment.

	Accuracy Summary	
Model id	Mean	Standard Deviation
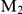	0.95	0.011
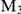	0.92	0.019
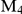	0.93	0.017

The accuracy summary for the cluster assignment is based on 50 simulated datasets under each model. The cluster assignment is estimated under the correct number of clusters.

**Figure 2:**
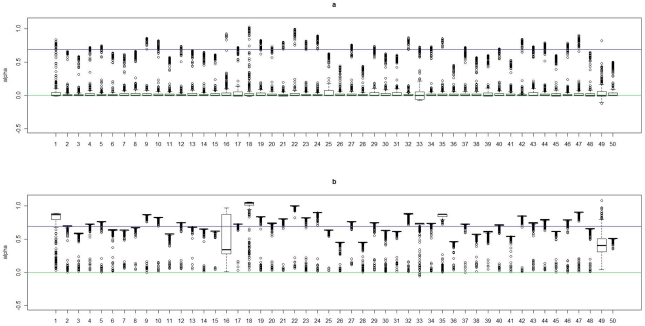
Boxplots of the posterior medians of the intercept (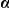) for subjects within each true cluster from each of 50 datasets simulated under the model 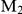. (a). Boxplots of posterior medians of 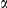 for subjects in cluster 1, with the true value given by the horizontal line in green; (b). Boxplots of posterior medians of 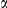 for subjects in cluster 2, with the true value given by the horizontal line in blue. The posterior median of 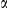 for each subject under a given simulated dataset was shifted by a constant value selected so that the median value of the shifted estimates for subjects in cluster 1 was zero.

**Figure 3:**
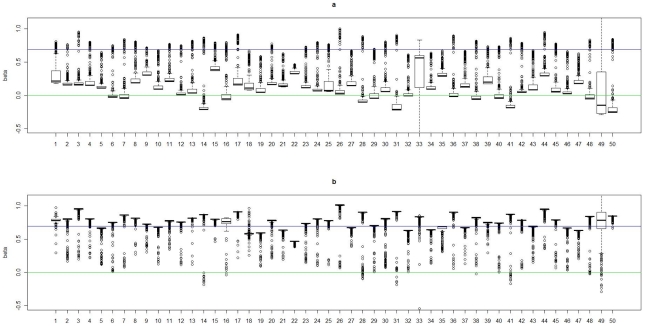
Boxplots of the posterior medians of the log odds ratio (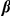) for subjects within each true cluster from each of 50 datasets simulated under the model 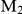. (a). Boxplots of posterior medians of 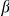 for subjects in cluster 1, with the true value given by the horizontal line in green; (b). Boxplots of posterior medians of 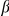 for subjects in cluster 2, with the true value given by the horizontal line in blue.

**Figure 4:**
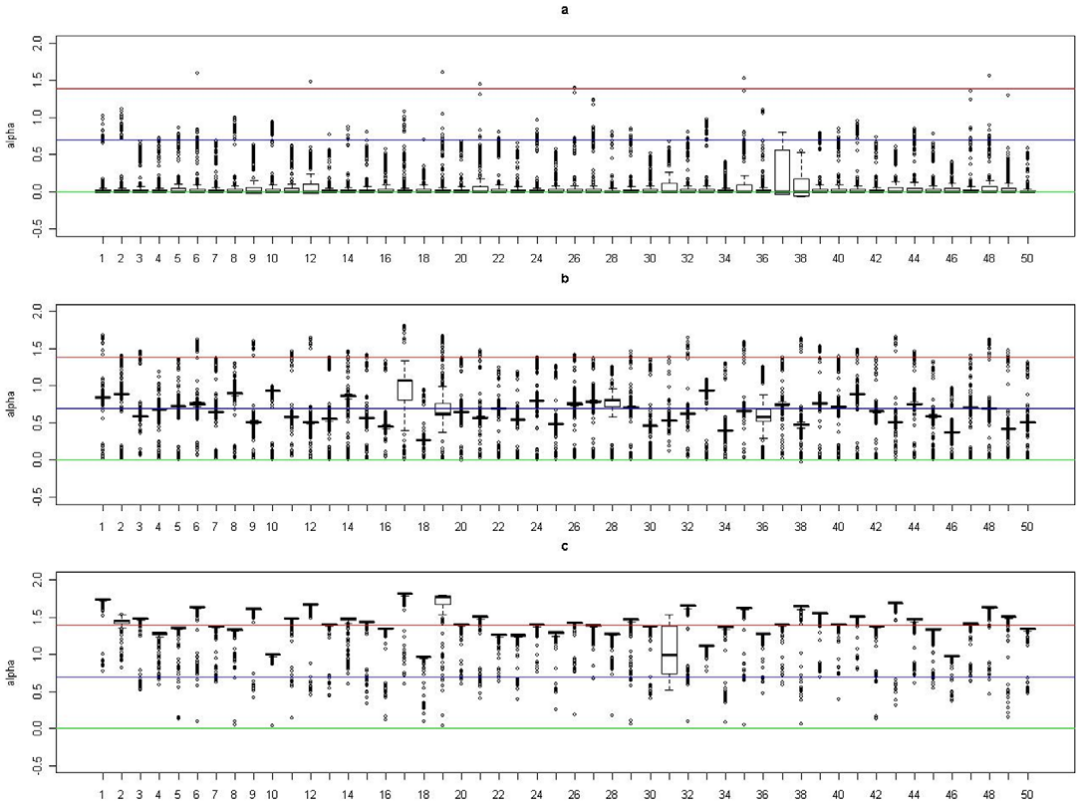
Boxplots of the posterior medians of the intercept (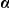) for subjects within each true cluster from each of 50 datasets simulated under the model 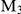. (a). Boxplots of posterior medians of 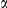 for subjects in cluster 1, with the true value given by the horizontal line in green; (b). Boxplots of posterior medians of 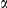 for subjects in cluster 2, with the true value given by the horizontal line in blue; (c). Boxplots of posterior medians of 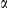 for subjects in cluster 3, with the true value given by the horizontal line in red. The posterior median of 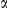 for each subject under a given simulated dataset was shifted by a constant value selected so that the median value of the shifted estimates for subjects in cluster 1 was zero.

**Figure 5:**
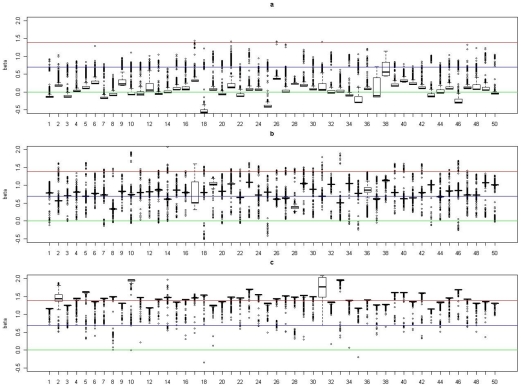
Boxplots of the posterior medians of the log odds ratio (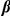) for subjects within each true cluster from each of 50 datasets simulated under the model 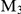. (a). Boxplots of posterior medians of 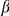 for subjects in cluster 1, with the true value given by the horizontal line in green; (b). Boxplots of posterior medians of 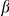 for subjects in cluster 2, with the true value given by the horizontal line in blue; (c). Boxplots of posterior medians of 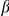 for subjects in cluster 3, with the true value given by the horizontal line in red.

We also evaluated the accuracy of the estimated coefficients (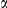 and 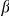). Based on the true risk model (1), subject *i* with genotype 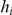 was assigned to one of the risk models. We considered coefficients 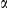 and 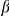 in that risk model to be the true coefficient values for this subject. Thus, subjects with their genotypes belonging to the same cluster would share the same true coefficient values. We used 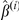, the posterior median of 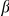 assigned to subject *i* based on MCMC samples generated under 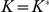, as the estimates for the underlying coefficients. The number of clusters 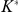 was estimated by the +1 rule, as described previously. Since the odds for the genetic effect is not identifiable under the case-control design, we were interested only in the difference in 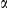 between two groups. To present the result for each experiment, we shifted the value of 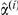, the posterior median of 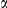 for subject *i*, by a constant value, which was chosen as 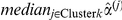, the median of 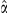 among subjects in true cluster 1. As a result, the median level of the shifted posterior median (we still represent it as 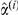) among subjects in cluster 1 is 0. In Figure 2, Figure 3, Figure 4, and Figure 5, we present summaries of 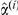 and 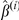 for each of the 50 experiments under models 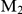 and 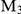. Summarized results for model 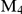 are given in Figures S1 and S2. Each boxplot is a summary of 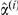 or 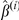 among subjects in a true underlying cluster. From those figures, we can see that the estimates align with their true values quite well. Notice that these estimates were obtained under the model with the number of clusters estimated by the +1 rule.

We inspected the algorithm's convergence using the Gelman and Rubin's diagnostic plot [33], as implemented in the CODA R package [34]. For each model, we checked the convergence on the first 5 simulated datasets used in the above simulation studies, with 5 independent runs on each dataset. We found that the proposed algorithm can converge within 100,000 iterations, with the estimated shrinkage factor falling below the recommended threshold of 1.1. We also show in Figures S3 and S4 the posterior distributions for 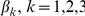, resulting from each of 5 independent runs on the first simulated dataset under models 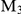, and 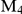. It is evident from these plots that we can obtain very consistent posterior distributions for parameters of interest among different runs on the same data.

### Simulation Studies: Performance of the Resampling-Based Test

We conducted a simulation study to evaluate whether the proposed resampling-based test can maintain the proper type I error rate. We considered a disease risk model that had the main effects from **G** (with OR = 4 for genotypes falling into regions II and III vs. those in region I) and *E* (with a common OR of 4 for 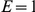 vs. 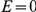), with no interaction between **G** and *E*. Regions are defined in Figure 1. We assumed a study sample size of 600 cases and 600 controls, and simulated 1000 datasets under the considered risk model as did before. For each dataset, we ran the resampling-based test with 1000 bootstrap steps for the estimation of the P-value, allowing the number of clusters to vary from 2 to 5. To reduce the computing time further, we ran the MCMC algorithm for 300,000 iterations with the burn-in period consisting of the first 200,000 iterations for each bootstrapped sample (as 200,000 iterations appear to be enough to ensure the convergence of the MCMC algorithm). We found that the proposed resampling-based test can maintain the proper type I error in the considered scenario, with estimated false positive rates of 0.055 and 0.097 under nominal levels of 0.05 and 0.10, respectively.

We compared the power of the proposed resampling-based test with two other standard interaction tests, the minP-SNP and minP-PC tests. Both test statistics are based on the minimum P-value observed on a set of univariate G-E interaction tests, with their significant levels being evaluated through a resampling-based procedure. The minP-SNP test is based on the set of single SNP-environment interaction tests, with each SNP-environment interaction test statistic being derived from the standard likelihood ratio test comparing two logistic regression models with and without the SNP-environment interaction term. The SNP effect is modeled with a categorical variable with three levels so each SNP-environment interaction test considered in the minP-SNP test is a 2 df test. The minP-PC is based on a set of tests that evaluate the interaction between a single principal component (PC) and the environment variable. PCs are derived from the principal component analysis of genotypes on all considered SNPs. Similar to the minP-SNP test, each PC-environment interaction test is derived from the likelihood ratio test comparing two logistic regression models with and without the interaction term. The PC effect is model as a continuous variable. Both minP-SNP and minP-PC were based on 15 univariate tests in the simulation study as there were a total of 15 SNPs in the considered chromosome region.

**Figure 6:**
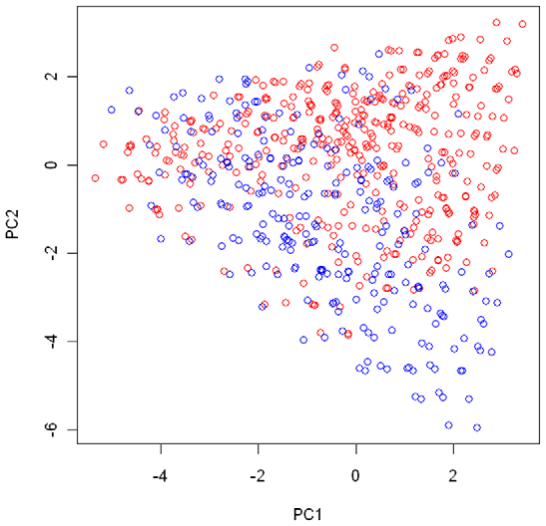
Cluster assignment for the EAGLE study. The cluster assignment estimated under the model with the number of clusters *K* = 2. Every subject was represented by his or her first 2 principal components. Subjects with the same multilocus genotype were represented by one point in the plot.

We evaluated the power under six different disease risk models, including 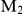, 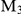, and 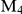 defined in Table 2, and the three additional models 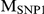, 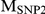, and 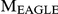. Model 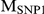 and 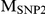 had just one functional SNP (the 10^th^ SNP in Table 1). Model 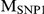 had 2 clusters, with coefficients in the formula (1) being 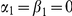 for genotypes satisfying the condition 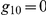 (cluster 1), and 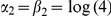 for 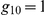, or 2 (cluster 2). Model 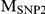 had 3 clusters, with coefficients 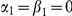 for 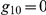 (cluster 1), 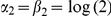 for 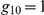 (cluster 2), and 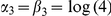 for 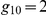 (cluster 3). Model 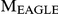 adopted a 2-cluster pattern observed in the analysis of the EAGLE study described later, with clusters 1 and 2 consisting of genotypes in red and blue colors, respectively (Figure 6), and with 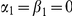 for cluster 1, and 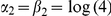 for cluster 2. The correlation between *E* and **G** was defined similarly as before. For a subject with genotype in cluster 1, the probabilities of being unexposed (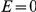) or exposed (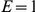) were 0.7 and 0.3; for a subject with genotype not in cluster 1, those probabilities were 0.4 and 0.6.

Under each disease model, we simulated 500 datasets, with each consisting of 600 cases and 600 controls. The summary for the power comparison results is given in Table 5. It can be seen from the table that the proposed test has a clear advantage over two other standard interaction tests, especially when the underlying clustering pattern in the disease risk cannot be properly approximated by a single SNP or PC (e.g., under the model 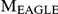). Even under the model 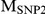 where the single SNP-environment interaction test based on the 10^th^ SNP is most optimal, due to the multiple comparison adjustment, the minP-SNP test is only slightly more powerful than the proposed test. Under the model 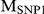 where the functional SNP (the 10^th^ SNP) has a dominant effect in its interaction with *E*, the minP-SNP test compares less favorably with the proposed test since each of single SNP-environment interaction test considered in the minP-SNP global test spends one more df than necessary (as there are only two cluster in the model 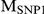). The minP-PC test has the worst overall performance as it is very sensitive to its underlying assumption that the genetic effect is linearly correlated with one of the PC direction.

**Table 5:** Power comparison under the type I error rate of 0.05.

	Power		
Risk Model	Proposed method	minP-SNP	minP-PC
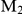	0.53	0.38	0.09
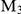	0.75	0.73	0.71
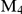	0.87	0.72	0.51
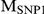	0.71	0.60	0.08
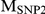	0.62	0.65	0.60
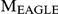	0.94	0.27	0.32

Risk models are defined in the main text. The power is estimated based on 500 simulated datasets, each consisting of 600 cases and 600 controls.

### Application in the EAGLE Study

We applied the proposed method to study the joint effect of cigarette smoking intensity (number of packs per day) and genetic variants in chromosome region 15q25.1 on lung cancer risk, using data generated by the EAGLE study. We focused on former and current smokers who had been genotyped on the 15 tagging SNPs. We also removed, as outliers, 8 subjects who had smoked more than 3 packs of cigarette per day. The final dataset for our analysis consisted of 1326 controls and 1720 cases. In the analysis we treated smoking intensity as a continuous variable and adjusted for the effects of gender and of age at diagnosis (categorized as: < = 60, 61–70, >70). We used a Bayesian model that allowed for **G**-*E* interaction, unless specified otherwise.

**Figure 7:**
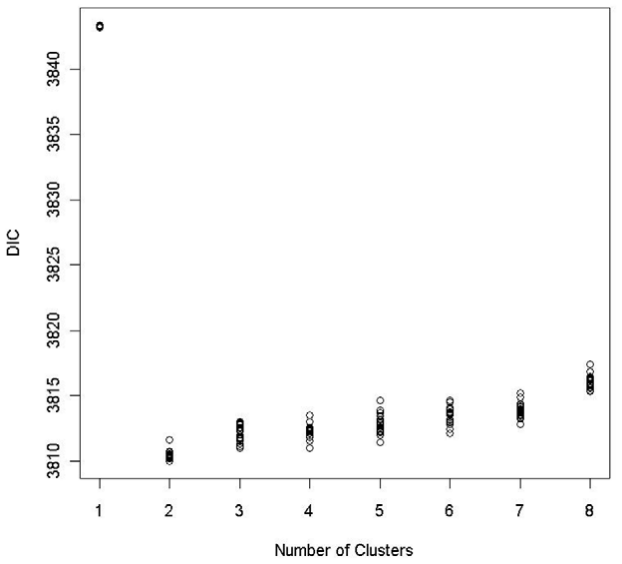
DIC plots for the Bayesian risk model allowing for gene–environment interaction. For any given number of clusters, 20 DIC values were obtained by applying the proposed method to the data from the EAGLE study 20 times with different random seeds.

To determine the number of clusters, we ran the MCMC algorithm 20 times with different random seeds for each *K*, 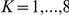, in order to estimate the Monte Carlo standard error for DIC. Figure 7 shows the DIC values for each *K* over 20 replications. Based on the 1 SE rule given by (7), the optimal number of clusters was found to be 2, with its averaged DIC value being 3810.5. The partitioning of subjects into 2 clusters based on our proposed clustering algorithm is very consistent among 20 replications. The discrepancy rate between assignments from any two runs, defined as the proportion of subjects being assigned to two different clusters, is less than 1.4% under 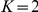.

Below we present the posterior summary based on a single run of our algorithm. To present the summary result, we first conducted a PC analysis on the case-control samples using genotypes at the 15 tagging SNPs as coordinates. In Figure 6, we plotted subjects by their first 2 PCs, with different colors representing their cluster assignments under 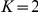. The cluster assignment was performed with the ensemble averaging method described above. Since subjects with the same genotype were represented by one point in the first 2-PC space, we can think of each point as either a unique genotype or a group of subjects sharing that genotype. There are 2240 subjects with 410 unique genotypes grouped into one cluster (shown in red in Figure 6) and 806 subjects with 252 unique genotypes grouped into another cluster (shown in blue in Figure 6). Notice that the two clusters are defined in term of estimated risk coefficient values (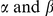), but not in term of genotypes distribution in the PC space. That is why these two clusters do not appear to be well separated in the PC space.

To summarize the effect of smoking on a subject with genotype *h*, 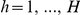, we focused on 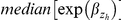, the posterior median of 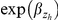, with 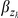 being the coefficient for smoking in the risk model assigned for a subject with genotype *h*. We can interpret 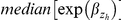 as the posterior median of the OR associated with one more pack of cigarettes per day for a subject with genotype *h*. To summarize the genetic effect of genotype *h*, we used 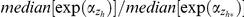, the ratio of the posterior median of 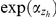 versus the posterior median of 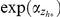, with 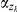 being the intercept for the risk model assigned for a subject with genotype *h* and 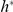 being the chosen reference genotype. We chose the reference genotype 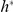 as the one having the lowest posterior median of 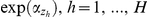. We can interpret 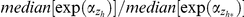 as the posterior median OR between genotype *h* and the reference genotype 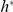.

**Figure 8:**
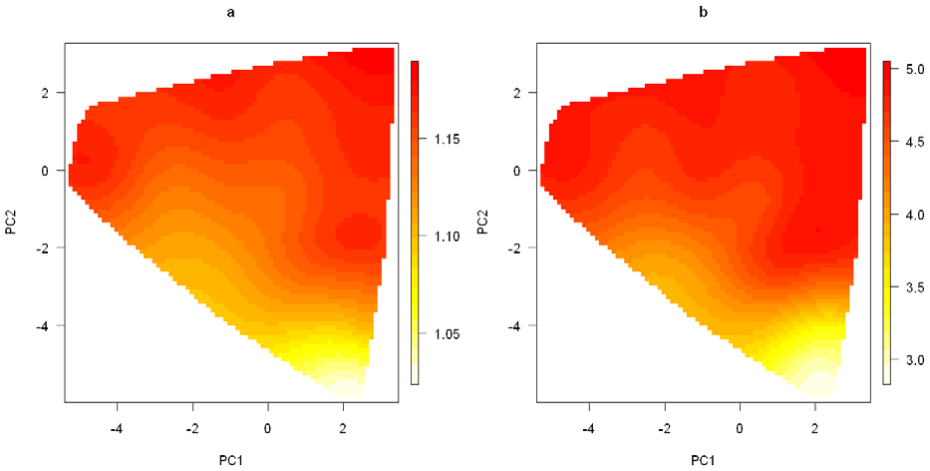
Smoothed surface plots of the posterior medians of the odds ratios for the genetic and smoking effects on the space of the first two principal components. (a). Posterior median of the OR for the genetic effect under the model with the number of clusters *K* = 2; (b). Posterior mean of the OR for the smoking effect under the model with the number of clusters *K* = 2.

In Figure 8, we show a smoothed surface plot for the smoking effect measured by 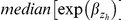, and the genetic effect measured by 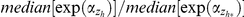 for each genotype in the first 2-PC space, based on models run under 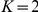. The smooth surface was estimated by the kriging method with each genotype's top 5 PCs (which account for over 85% of the total variation) as predictors. The plots were generated using the functions provided in the R package called “fields” [35]. It is evident from Figure 8 that neither the smoking effect nor the genetic effect is uniformly distributed over the genotype space. The smoking effect on a subject depends on his or her genotype. It is considerably lower on subjects who have their genotypes projected on the lower part of the PC space than on subjects with their genotypes projected elsewhere.

Some understanding of the 2^nd^ PC is helpful for interpreting the patterns observed in Figure 8. From Table 1, we can see that the 2^nd^ PC is driven mainly by the 8 SNPs with absolute loading values larger than 0.18, with the remaining having loading values less than 0.07. These 8 SNPs also turn out to be the ones that are most significantly associated with lung cancer risk (Table 1). We point out the fact that the loading value for each of the 8 SNPs is negative if the SNP's major allele is the high-risk allele, positive if its minor allele is the high-risk allele. As a result, a genotype's 2^nd^ PC coordinate is positively correlated with its total number of risk alleles among the 8 SNPs (see Figure S5). Genotypes with smaller 2^nd^ PC coordinates tend to have fewer high-risk alleles and are expected to have smaller ORs than genotypes having larger 2^nd^ PC coordinates.

As a comparison, we also fit model (3), the Bayesian model without **G**-*E* interaction. The optimal model based on the 1 SE rule was again achieved at 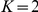, with its averaged DIC value being 3817.5 over 20 runs (Figure S6). The DIC is noticeably higher than that obtained under the Bayesian model allowing for **G**-*E* interaction (DIC = 3810.5). This suggests that the model allowing for **G**-*E* interaction fits the data better than the model without the **G**-*E* interaction.

Finally, to demonstrate the existence of **G**-*E* interaction further, we applied the resampling-based test described in the Methods section. The observed test statistic was 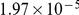. We applied the resampling-based test by allowing the number of clusters to vary from 2 to 5. The estimated P-value based on 2000 bootstrap steps was 0.016, suggesting that there is a significant **G**-*E* interaction. On the other hand, for each of the 32 relatively common SNPs (MAF>0.04) in this considered 15q25.1 region, we conducted the standard SNP-smoking interaction test (2 df) based on the logistic regression model by treating the genotype as a three-level categorical variable. The smallest nominal P-value we observed was 0.021. The global minP-SNP test had a P-value of 0.29, which was well above the 0.05 level. We also conducted the PC-smoking interaction test by modeling each PC as a continuous variable. The smallest nominal P-value was 0.058. The P-value from the global minP-PC test was 0.62.

## Discussion

Our new method can evaluate gene–environment interaction at the gene/region level by integrating information observed on multiple SNPs in the considered gene/region with measures of environmental exposure. This method reduces the impact of loss of efficiency and bias from the misclassification error inherent in the single-marker approach that studies the environmental risk factor and one SNP at a time. The method provides a coherent inference framework that allows us to evaluate the environmental effect on different strata defined by the multi-locus genotype. A heterogeneous environmental effect across different strata would signal the presence of gene–environment interaction. We also propose a resampling-based test to formally test for the existence of gene–environment interaction.

Genetic variations within the 15q25.1 region have been shown to be associated with both lung cancer risk and smoking behaviors, such as the smoking intensity. Our analysis based on the EAGLE case-control study indicates that the smoking effect varies according to the subject's genetic makeup in the 15q25.1 region. The proposed resampling-based test also supports the existence of gene–environment interaction (P-value = 0.016). On the other hand, two conventional tests of gene–environment interaction based on the single-marker and single-PC approaches are far from significant. This highlights the advantage of our proposed method over standard approaches.

Accurate assessment of the environment risk exposure in the evaluation of gene–environment interaction is as important as identification of functional genetic variants or their proper surrogates [36]. In the EAGLE population-based case-control study, the information on smoking collected near the time of diagnosis is likely to provide a more accurate measure of risk exposure than information collected in other prospective cohort studies, such as the Prostate, Lung, Colorectal, and Ovarian (PLCO) Screening Trial [37], which does not reflect subsequent changes in smoking behavior like quitting. For example, we observed a much larger OR for smoking one more pack of cigarette per day (3.7, *z* statistic = 15.58) in the EAGLE study than in a lung cancer case-control study nested within the PLCO cohort (1.84, *z* statistic = 8.87), which includes 1390 lung cancer cases and 1924 controls. We also could not find evidence for smoking-15q25.1 interaction in this PLCO nested case-control study by using our proposed method. The difference in the smoking OR estimates and the absence of gene–environment interaction evidence using our method in the PLCO study may be a consequence of greater misclassification error in the smoking information assessment in the cohort study (PLCO) than in the case-control study (the EAGLE study).

In our method, we adopted the Potts model for the latent allocation vector for cluster assignment, as did Green and Richardson [6]. We used the MCMH algorithm [12] for simulating the regulating parameter of the Potts model. The MCMH algorithm overcomes the intractable normalizing constant problem that cannot be handled by the standard MH algorithm, while ensuring the consistency of the Monte Carlo estimates. Furthermore, this MCMH algorithm can readily be used for Potts models with certain restrictions on the sampling space by modifying the MH step to generate allocation vectors accordingly.

We proposed to use the +1 SE rule (or the +1 rule) based on DIC to identify the optimal number of clusters. We found through simulation studies that this approach works quite well. An alternative approach would be to treat the number of clusters as a random variable and integrate it into a Bayesian model [6]. A reversible jump MCMC algorithm [38] could be used to facilitate the move between sampling spaces with different dimensions. It would be interesting to compare the performance of these approaches, especially in term of their abilities to identify the proper number of clusters.

The proposed procedure relies upon a user-specified similarity metric to define the neighborhood among different genotypes in the Potts model. This neighborhood structure is used to induce the spatial dependency in the cluster assignment. In this paper, for a given genotype, we chose its 4 nearest genotypes as its neighbors. We found that the analysis result was not very sensitive to how the neighborhood is defined as long as the chosen Markov structure can generate an appropriate spatial dependence. For example, we reanalyzed the EAGLE study with two other types of Markov structures: one using the 3 nearest genotypes as neighbors, and the other one using the 5 nearest genotypes as neighbors. We show in Figure S7 the comparison of the posterior medians of the genetic effect (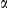) and the smoking effect (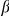) estimated for each subject between each of the new runs and the original runs under 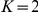. It is clear that results from these three analyses are quite similar.

We used the prospective likelihood model in the Bayesian framework for case-control studies, even though the data were collected retrospectively according to a subject's disease status. According to [23], [39], given certain priors for parameters in the retrospective model, we can derive corresponding priors for the prospective model parameters that yield the same marginal posterior distributions as their retrospective counterparts. In this paper we consider both normal and uniform distributions as priors for the prospective model parameters. Although we cannot derive explicitly their corresponding priors for the retrospective model, our simulation studies demonstrated that the proposed prospective approach indeed had the desired performance when applying to data generated from case-control studies. The normal prior has also been used with the prospective likelihood model on case-control studies in other contexts (e.g., [40], [41]).

We have created an R package called BaDGE (Bayesian model for Detecting Gene Environment interaction) implementing the proposed Bayesian model and the associated post-processing procedures. The package is freely available from the website http://dceg.cancer.gov/bb/tools/badge. Currently, we consider only binary or continuous environmental exposure variable. It is straightforward to expand the algorithm to deal with a categorical (with more than 2 levels) environmental variable. To use the program, the user needs to specify priors (normal or uniform distribution) for parameters in the risk model and a prior (a uniform distribution) for the regulating parameter in the Potts model. The program will be expanded further to incorporate other prior functions. The running time for 200,000 iterations using 50 auxiliary samples in the MCMH algorithm on a dataset of 1000 cases and 1000 controls, with approximate 450 unique genotypes, is about 14 minutes on a Linux machine with the 2.8 GHz AMD Opteron processor. For a dataset with a large number of genotypes (e.g., over 1000), we can reduce the computing time by first dividing the whole genotype space into a few hundreds of subgroups through the PAM clustering algorithm [30] and then treating subgroups as genotypes in the proposed MCMC procedure. For example, the running time on the same testing example mentioned above decreases to 8 minutes if we regroup the genotypes into 250 unique subgroups. Another way to reduce the total number of genotypes is to limit tagging SNPs to those with a relatively large minor allele frequency. The resampling-based test could be computationally intensive for a dataset like the EAGLE study. We are still investigating whether it is possible to replace the computationally intensive resampling-based procedure with a one-step multiple comparison adjustment approach, similar to one used in [42], for the assessment of the statistical significance level.

Comparing to the standard single-marker or principal component based approaches, our proposed method is more computationally intensive, but it has several important advantages. First, it offers a more flexible way to model gene–environment interaction, especially complicated ones that cannot be depicted properly by the single-marker or PC based approaches, such as in situations where genetic variants (might or might not be directly genotyped) in multiple loci within the considered region interplay with the environment risk factor. Second, it provides an estimate of the environmental effect on subjects with a given joint genotype profile. This could be potentially useful to generate new hypotheses on how the gene and environment risk factor interacts. Third, as shown in the simulation studies and real application, the proposed resampling-based test derived from the Bayesian model has a more robust performance than the standard single-marker, or PC based testing procedures. For example, in situation where the single marker test is most appropriate, i.e., there is only one functional locus in the considered region, the proposed test is only slightly less powerful than the single-marker test. But it has a considerable power advantage over the standard tests when the underlying disease risk pattern cannot be properly approximated by a single SNP or PC.

Although our method is described in the context of gene–environment interaction detection, it is in fact quite general. It provides a general strategy for studying the interaction between an observed risk factor and a latent categorical variable not directly observed or clearly defined, but one that can be derived from a set of observed relevant covariates. For example, our method can be used with some minor modifications to evaluate the interaction between smoking behavior (e.g., smoking intensity) and a latent dietary pattern that can be derived from food frequency questionnaires. Also, it is possible to extend our method to study gene-gene interaction by introducing two latent factors to capture the effect of both genes, as was done in [43].

## Supporting Information

Figure S1Boxplots of the posterior medians of the intercept (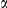) for subjects within each true cluster from each of 50 datasets simulated under the model 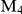. (a). Boxplots of posterior medians of 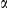 for subjects in cluster 1, with the true value given by the horizontal line in green; (b). Boxplots of posterior medians of 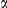 for subjects in cluster 2, with the true value given by the horizontal line in red; (c). Boxplots of posterior medians of 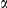 for subjects in cluster 3, with the true value given by the horizontal line in red. The posterior median of 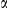 for each subject under a given simulated dataset was shifted by a constant value selected so that the median value of the shifted estimates for subjects in cluster 1 was zero.(TIF)

Figure S2Boxplots of the posterior medians of the log odds ratio (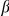) for subjects within each true cluster from each of 50 datasets simulated under the model 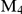. (a). Boxplots of posterior medians of 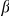 for subjects in cluster 1, with the true value given by the horizontal line in green; (b). Boxplots of posterior medians of 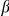 for subjects in cluster 2, with the true value given by the horizontal line in green; (c). Boxplots of posterior medians of 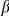 for subjects in cluster 3, with the true value given by the horizontal line in red.(TIF)

Figure S3Posterior distribution comparison among 5 independent runs under the model 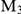. Plot *i*−*j* is the posterior distribution summary for the coefficient 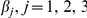, based on the *i*th, 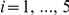, independent run on a dataset simulated under the model 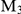.(TIF)

Figure S4Posterior distribution comparison among 5 independent runs under the model 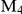. Plot *i*−*j* is the posterior distribution summary for the coefficient 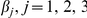, based on the *i*th, 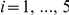, independent run on a dataset simulated under the model 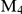.(TIF)

Figure S5 The correlation between the total number of risk alleles and the 2^nd^ principal components. Each point represents a unique multilocus genotype with its x-coordinate being the total number of risk alleles among those SNPs with high loading values (highlighted in Table 1 at the PC2 column) on the 2^nd^ principal components, its y-coordinate being the 2^nd^ principal component.
(TIF)

Figure S6 DIC plots for the Bayesian risk model without gene–environment interaction. For a given number of clusters, 20 DIC values were obtained by applying the model to the EAGLE study 20 times with different random seeds.
(TIF)

Figure S7Pairwise correlations of estimates by the algorithm with different neighborhood structures. The MCMC procedure was applied to the EAGLE study using three different Markov structures, M1: using the 3 nearest genotypes as neighbors; M2: using the 4 nearest genotypes as neighbors; and M3: using the 5 nearest genotypes as neighbors. (a) Comparison of the estimated genetic effect (in term of the posterior median of 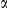) on each subject between the method using M1 and the one using M2; (b) Comparison of the estimated genetic effect between the method using M3 and the one using M2; (c) Comparison of estimated smoking effect (in term of the posterior median of 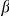) on each subject between the procedure using M2 and the one using M1; and (d) Comparison of the estimated smoking effect between the method using M3 and the one using M2.(TIF)

Text S1 MCMC algorithm details.
(DOC)
