# Supplementary figures and images for: A Flexible Bayesian Model for Studying Gene–Environment Interaction
Source: PLoS Genet. 2012 Jan 26;8(1):e1002482. doi: 10.1371/journal.pgen.1002482 (PMC3266891; doi:10.1371/journal.pgen.1002482)

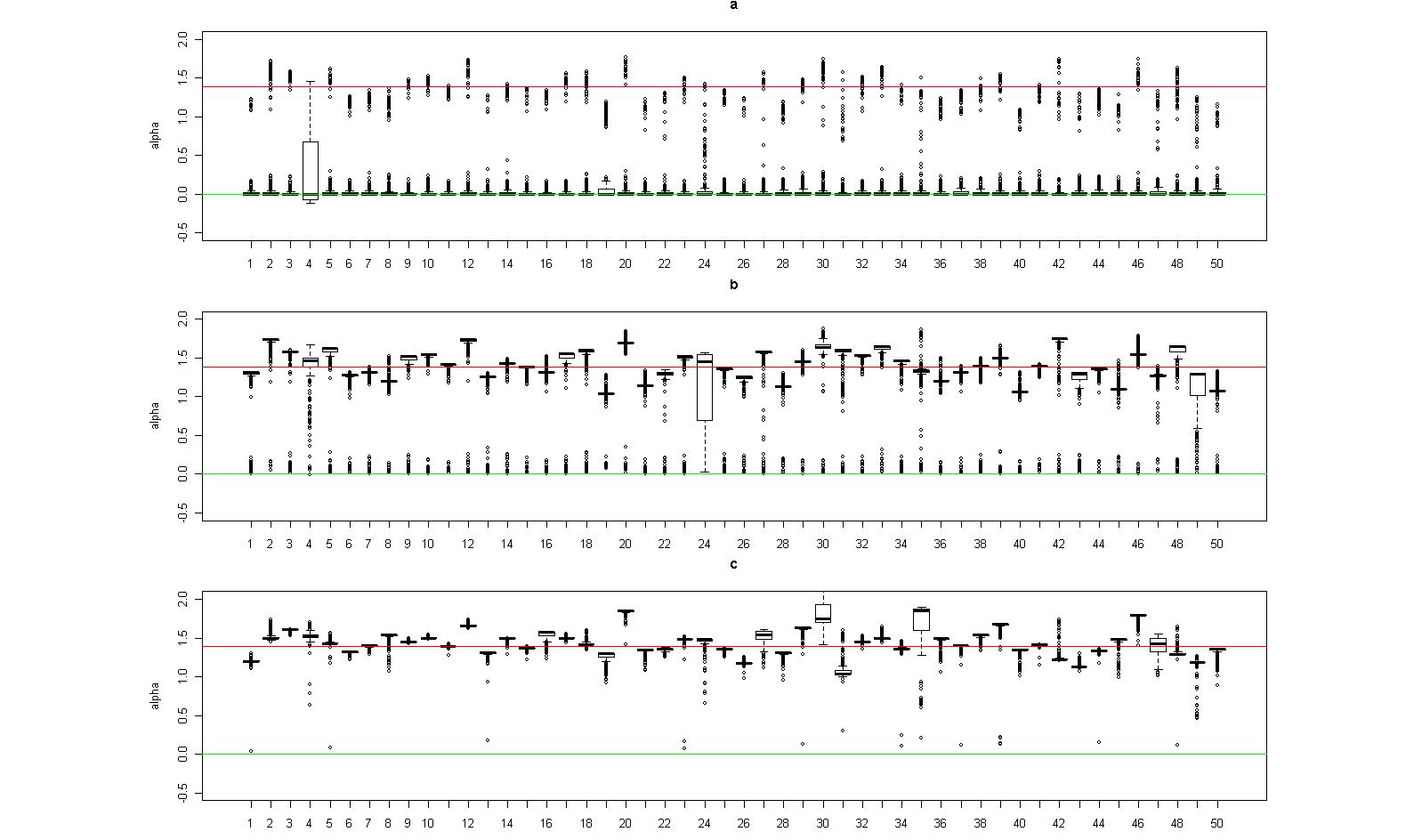

Supplement: Figure S1 — Boxplots of the posterior medians of the intercept () for subjects within each true cluster from each of 50 datasets simulated under the model . (a). Boxplots of posterior medians of for subjects in cluster 1, with the true value given by the horizontal line in green; (b). Boxplots of posterior medians of for subjects in cluster 2, with the true value given by the horizontal line in red; (c). Boxplots of posterior medians of for subjects in cluster 3, with the true value given by the horizontal line in red. The posterior median of for each subject under a given simulated dataset was shifted by a constant value selected so that the median value of the shifted estimates for subjects in cluster 1 was zero. (TIF) [file pgen.1002482.s001.tif]

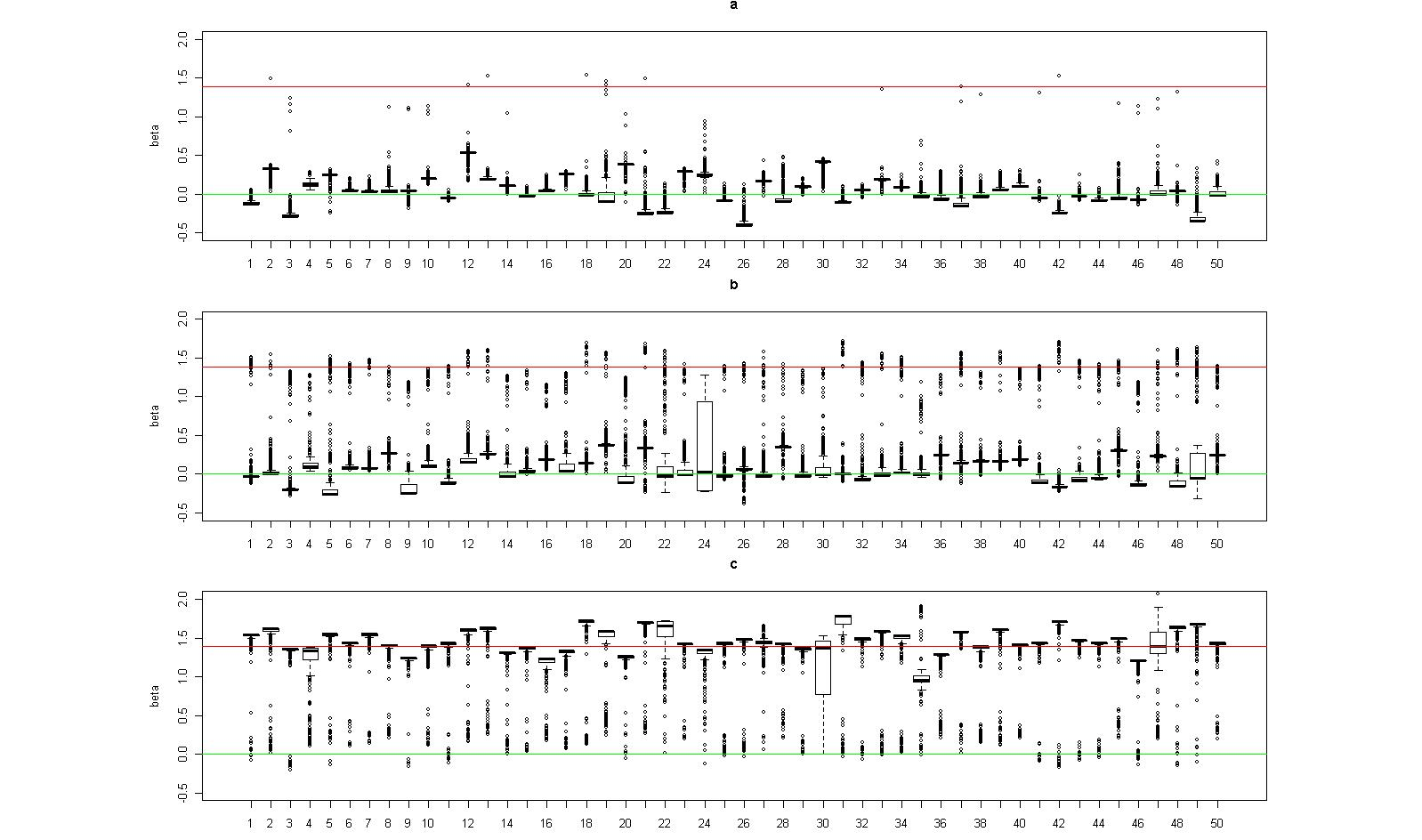

Supplement: Figure S2 — Boxplots of the posterior medians of the log odds ratio () for subjects within each true cluster from each of 50 datasets simulated under the model . (a). Boxplots of posterior medians of for subjects in cluster 1, with the true value given by the horizontal line in green; (b). Boxplots of posterior medians of for subjects in cluster 2, with the true value given by the horizontal line in green; (c). Boxplots of posterior medians of for subjects in cluster 3, with the true value given by the horizontal line in red. (TIF) [file pgen.1002482.s002.tif]

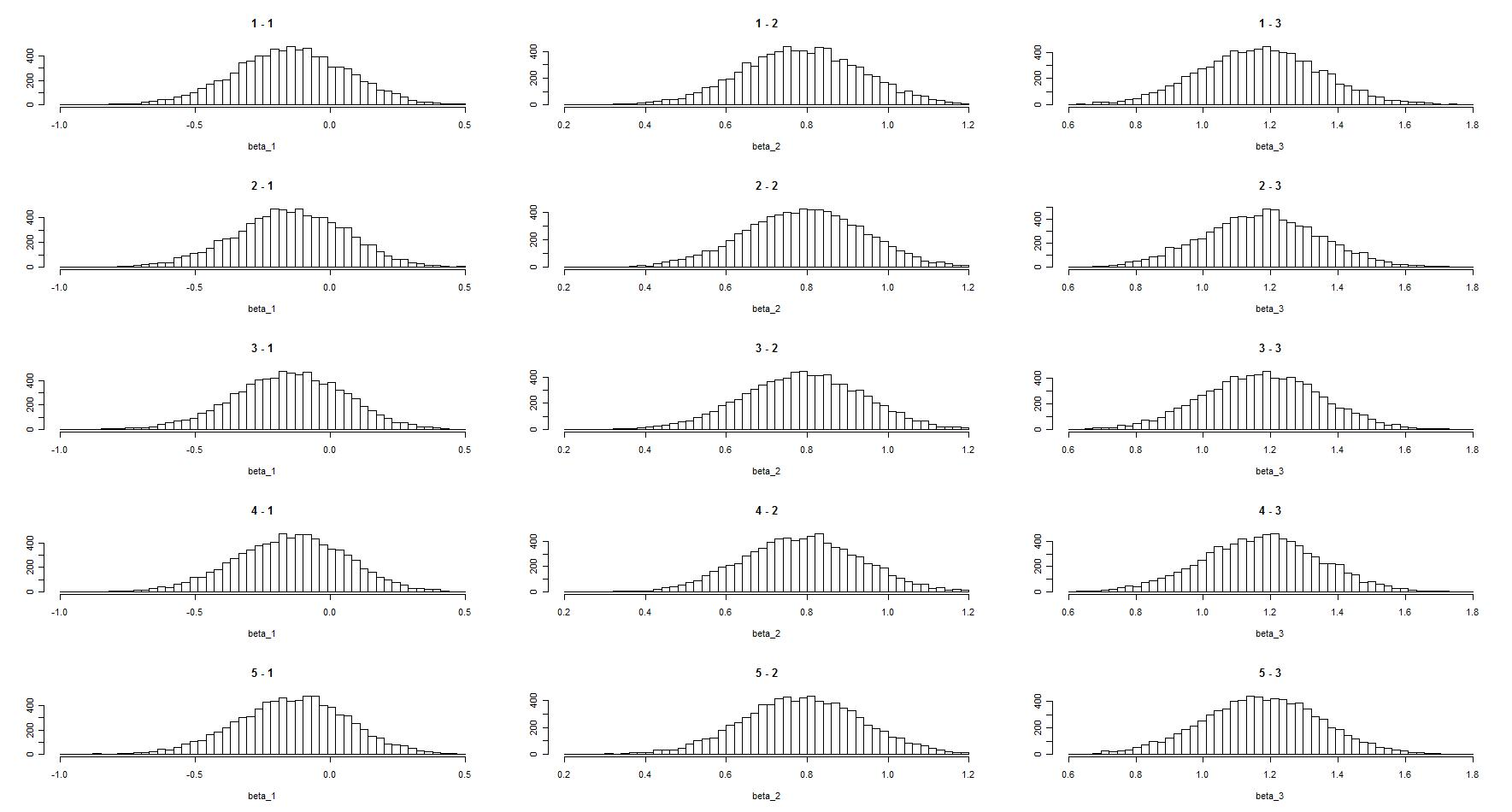

Supplement: Figure S3 — Posterior distribution comparison among 5 independent runs under the model . Plot i−j is the posterior distribution summary for the coefficient , based on the ith, , independent run on a dataset simulated under the model . (TIF) [file pgen.1002482.s003.tif]

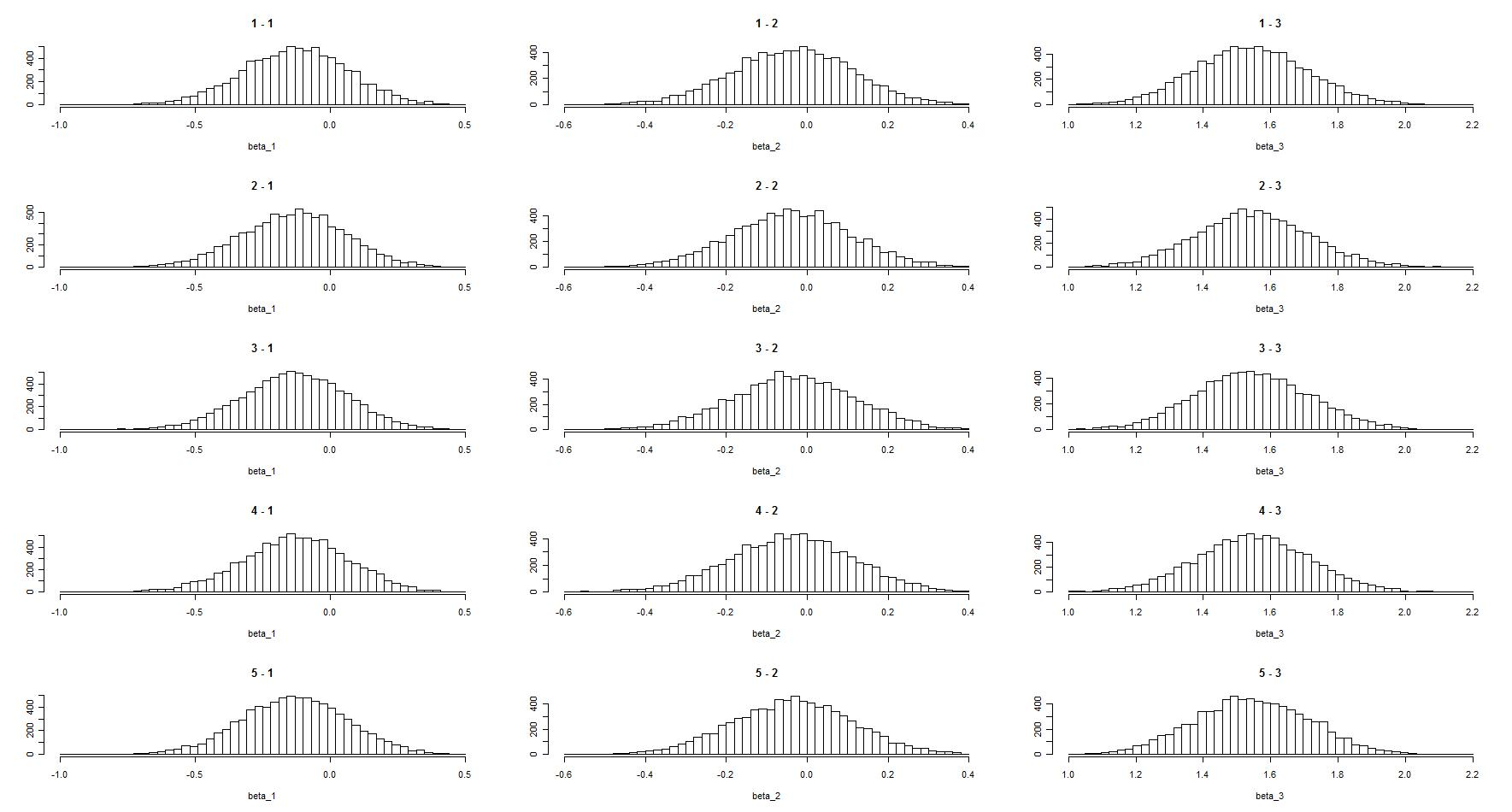

Supplement: Figure S4 — Posterior distribution comparison among 5 independent runs under the model . Plot i−j is the posterior distribution summary for the coefficient , based on the ith, , independent run on a dataset simulated under the model . (TIF) [file pgen.1002482.s004.tif]

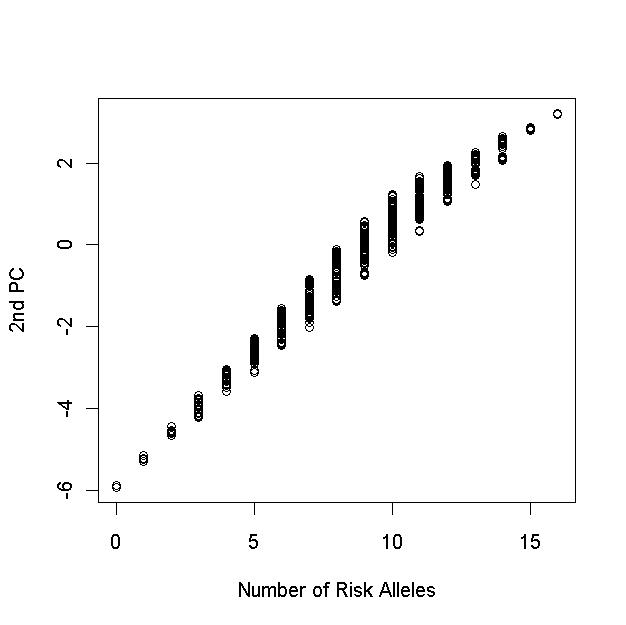

Supplement: Figure S5 — The correlation between the total number of risk alleles and the 2nd principal components. Each point represents a unique multilocus genotype with its x-coordinate being the total number of risk alleles among those SNPs with high loading values (highlighted in Table 1 at the PC2 column) on the 2nd principal components, its y-coordinate being the 2nd principal component. (TIF) [file pgen.1002482.s005.tif]

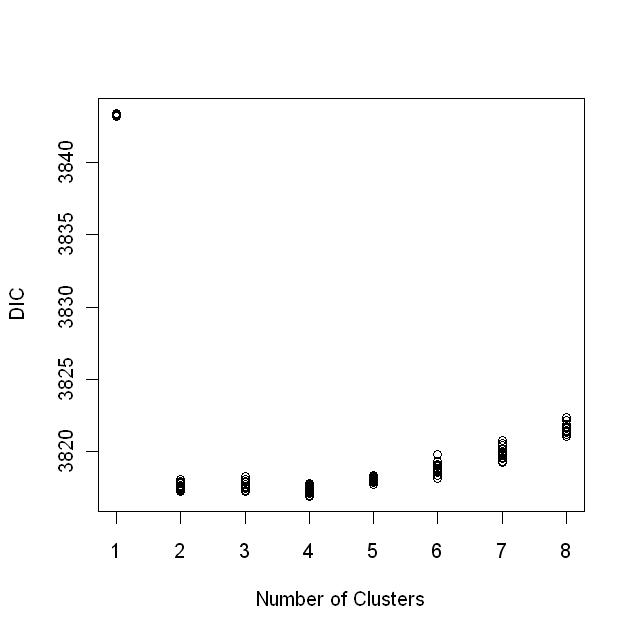

Supplement: Figure S6 — DIC plots for the Bayesian risk model without gene–environment interaction. For a given number of clusters, 20 DIC values were obtained by applying the model to the EAGLE study 20 times with different random seeds. (TIF) [file pgen.1002482.s006.tif]

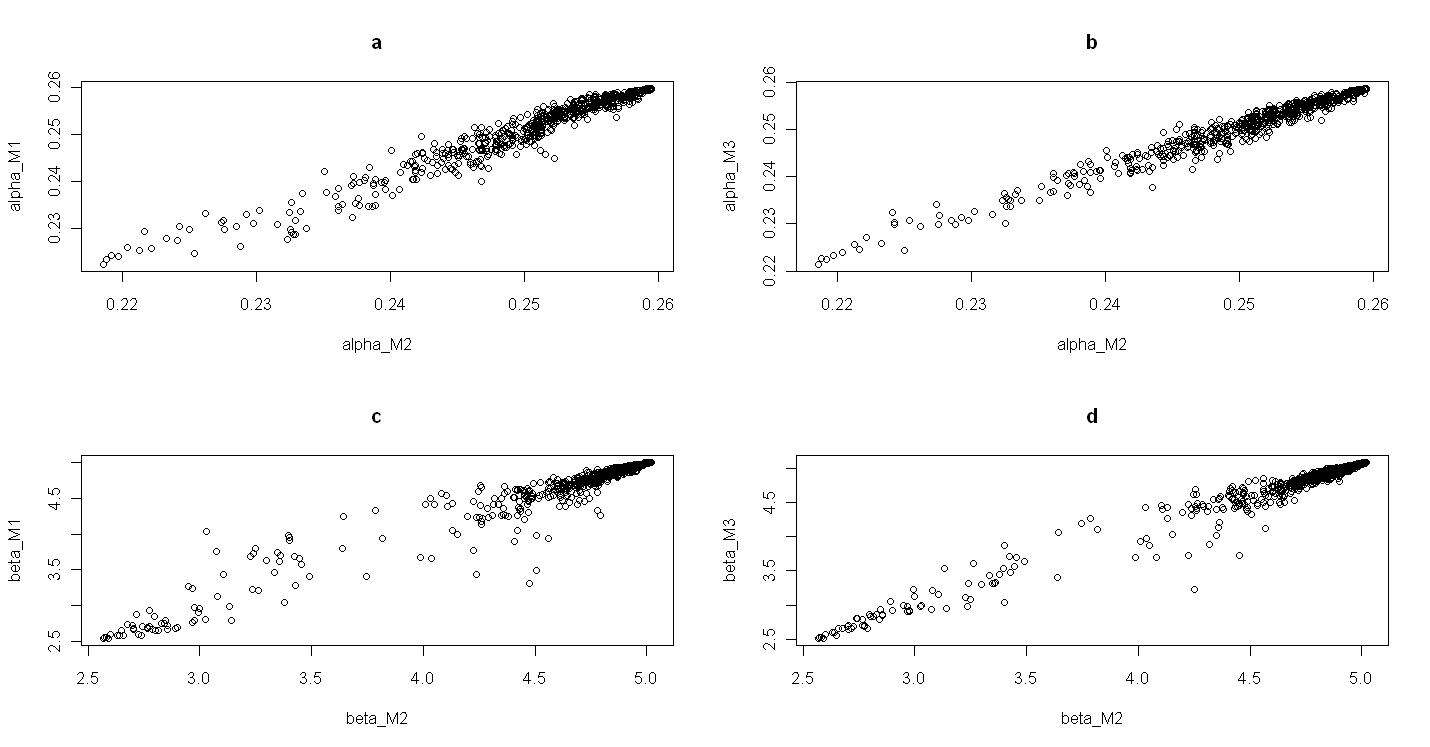

Supplement: Figure S7 — Pairwise correlations of estimates by the algorithm with different neighborhood structures. The MCMC procedure was applied to the EAGLE study using three different Markov structures, M1: using the 3 nearest genotypes as neighbors; M2: using the 4 nearest genotypes as neighbors; and M3: using the 5 nearest genotypes as neighbors. (a) Comparison of the estimated genetic effect (in term of the posterior median of ) on each subject between the method using M1 and the one using M2; (b) Comparison of the estimated genetic effect between the method using M3 and the one using M2; (c) Comparison of estimated smoking effect (in term of the posterior median of ) on each subject between the procedure using M2 and the one using M1; and (d) Comparison of the estimated smoking effect between the method using M3 and the one using M2. (TIF) [file pgen.1002482.s007.tif]
