# Supplementary material for: A Flexible Bayesian Model for Studying Gene–Environment Interaction
Source: PLoS Genet. 2012 Jan 26;8(1):e1002482. doi: 10.1371/journal.pgen.1002482 (PMC3266891; doi:10.1371/journal.pgen.1002482)
Supplement: Text S1 — MCMC algorithm details. (DOC) [file pgen.1002482.s008.doc]

**Text S1. MCMC algorithm details**

1. ***Algorithm for updating*** $\Gamma$

For notational simplicity, we assume that the dimension of the adjusted covariate $\tau$ is 1. Define $\alpha=\left(\alpha_{1},...,\alpha_{K}\right)^{'}$, $\beta=\left(\beta_{1},...,\beta_{K}\right)^{'}$, and $\tau$as the current version of the coefficients. Here is a summary of the algorithm.

1. Generate two independent vectors from the *K*-dimensional multi-normal distribution with $\left(0,...,0\right)^{'}$ as mean and the identity matrix as the covariance matrix, and standardize them so that each has unit 1. Denote the two unit vectors as $\mathbf{e}_{\alpha}$ and $\mathbf{e}_{\beta}$.
2. Generate $\xi_{\alpha}\sim N(0,\sigma_{\alpha}^{2})$, $\xi_{\beta}\sim N(0,\sigma_{\beta}^{2})$, and $\xi_{\tau}\sim N(0,\sigma_{\tau}^{2})$, with $\sigma_{\alpha}^{2}$, $\sigma_{\beta}^{2}$, and $\sigma_{\tau}^{2}$ specified by the user.
3. Given $\left(\alpha,\beta,\tau\right)$, obtain their proposed update as $\alpha^{*}=\alpha+\xi_{\alpha}\mathbf{e}_{\alpha}$, $\beta^{*}=\beta+\xi_{\beta}\mathbf{e}_{\beta}$, and $\tau^{*}=\tau+\xi_{\tau}$.
4. Update the coefficients as $\left(\alpha^{*},\beta^{*},\tau^{*}\right)$ with probability $\min\left(1,r\right)$, and keep their current values unchanged with probability $1-\min\left(1,r\right)$, where $r=p(\Gamma^{*}|\cdots)/p(\Gamma|\cdots)$, with $\Gamma^{*}=\left(\alpha^{*},\beta^{*},\tau^{*}\right)$ and $p(\Gamma|\cdots)$ given by (4).

In the implementation, we can choose $\sigma_{\alpha}^{2}$, $\sigma_{\beta}^{2}$, and $\sigma_{\tau}^{2}$ around the same level as estimated variances of the MLEs for the corresponding coefficients in a logistic regression model with only *E* and *X*.

***II. Algorithm for updating* z**

Let $\mathbf{z}^{(t)}=\left(z_{1}^{(t)},...,z_{H}^{(t)}\right)$ be the current version of the allocation vector. Generate $\mathbf{z}^{(t+1)}=\left(z_{1}^{(t+1)},...,z_{H}^{(t+1)}\right)$ by repeating the following steps for $h=1,\text{ ..., }H$, starting with $\mathbf{z}^{(t+1)}=\mathbf{z}^{(t)}$ :

1. Randomly pick a cluster id other than $z_{h}^{(t+1)}$, and denote it by $k^{*}$.
2. Let $z_{h}^{(t+1)}=k^{*}$ with probability min(1, *r*), and leave $z_{h}^{(t+1)}$ unchanged with probability $1-\min(1,r)$, where $r=\frac{p(z_{h}=k^{*}|...)}{p(z_{h}=z_{h}^{(t+1)}|...)}$, with $p(z_{h}|...)$given by (5).

Note that in the calculation of *r* in step 2, elements conditioned upon include the current version of $\mathbf{z}^{(t+1)}$.

***III. Algorithm for updating*** $\psi$

To use the MCMH algorithm, we first need to draw auxiliary samples from the Potts model $p(\mathbf{z}|\psi)\propto\exp\left[\psi U(\mathbf{z})\right]$, given $\psi$, with $\mathbf{z}\in\left(1,...,K\right)^{H}$. Here is the procedure to generate *m* (e.g., $m=50$) copies of allocation vectors (auxiliary samples) at the beginning of the whole MCMC algorithm, with a starting allocation vector $\mathbf{w}^{(0)}$. Notice that these auxiliary samples are completely independent from the allocation vector **z** generated in the MCMC steps. Let $\psi_{0}$ be the current value for the regulating parameter $\psi$. Given $\mathbf{w}^{(a-1)}=\left(w_{1}^{(a-1)},...,w_{H}^{(a-1)}\right)$, we generate the next auxiliary sample $\mathbf{w}^{(a)}$, $a=1,\text{ ..., }m$, by repeating the following Metropolized Gibbs steps for $h=1,\text{ ..., }H$, starting with $\mathbf{w}^{(a)}=\left(w_{1}^{(a-1)},...,w_{H}^{(a-1)}\right)$.

1. Randomly pick a cluster id other than $w_{h}^{(a)}(=k)$, and denote it by $k^{*}$.
2. Let $w_{h}^{(a)}=k^{*}$ with probability min(1, *r*), and leave $w_{h}^{(a)}$ unchanged with probability $1-\min(1,r)$, where $r=\exp\left\{\psi\left[t_{h}^{k^{*}}\left(\mathbf{w}^{(a)}\right)-t_{h}^{k}\left(\mathbf{w}^{(a)}\right)\right]\right\}$, with $t_{h}^{k}(\cdot)$ defined as in (5) in the main text.

Once we have the initial auxiliary samples, we can use the following MCMH steps to update $\psi$:

1. Let $\psi$ denote the current value for the regulating parameter, and let $\mathbf{w}^{(1)},\mathbf{w}^{(2)},...,\mathbf{w}^{(m)}$ be the current set of auxiliary samples.
2. Generate $\xi_{\psi}\sim N(0,\sigma_{\psi}^{2})$, with $\sigma_{\psi}^{2}$ specified by the user. Let $\psi'=\psi+\xi_{\psi}$.
3. If $\psi'$is not in the supporting region specified by the uniform prior, then $\psi$ remains the same, as do $\mathbf{w}^{(1)},\mathbf{w}^{(2)},...,\mathbf{w}^{(m)}$.
4. If $\psi'$falls into the supporting region specified by the uniform prior, do the following:

4.1. Estimate the normalizing constant ratio by

$\hat{R}(\psi,\psi')=\frac{1}{m}\sum_{a=1}^{m} \exp\left[(\psi'-\psi)U(\mathbf{w}^{(a)})\right]$ .

4.2. Calculate the MH ratio $r=\frac{\exp\left[(\psi'-\psi)U(\mathbf{z})\right]}{\hat{R}(\psi,\psi')}$.

4.3. Set $\psi=\psi'$ with probability $\min(1,r)$, and keep $\psi$ unchanged with probability $1-\min(1,r)$.

4.4. If $\psi$ is updated to a new value $\psi'$, we also update the auxiliary samples $\mathbf{w}^{(1)},\mathbf{w}^{(2)},...,\mathbf{w}^{(m)}$ using the following procedure.

***Algorithm for updating auxiliary samples*:**

1. For each $\mathbf{w}^{(a)},\text{ }a=1,...,m,$ obtain its weight $\omega_{a}=\exp\left[(\psi'-\psi)U(\mathbf{w}^{(a)})\right]$.
2. Generate a new $\mathbf{w}^{(0)}$ by randomly drawing with replacement from $\mathbf{w}^{(1)},\mathbf{w}^{(2)},...,\mathbf{w}^{(m)}$ according to the distribution $\left(\omega_{1},...,\omega_{m}\right)$.
3. Once we have the new $\mathbf{w}^{(0)}$**,** use the algorithm for initializing the auxiliary samples to generate a new set of $\mathbf{w}^{(1)},\mathbf{w}^{(2)},...,\mathbf{w}^{(m)}$ with $\psi_{0}=\psi'$.
